# Editorial: Microbiota of Grapes: Positive and Negative Role on Wine Quality

**DOI:** 10.3389/fmicb.2016.02036

**Published:** 2016-12-21

**Authors:** Giuseppe Spano, Sandra Torriani

**Affiliations:** ^1^Department of the Sciences of Agriculture, Food and Environment, University of FoggiaFoggia, Italy; ^2^Department of Biotechnology, University of VeronaVerona, Italy

**Keywords:** grape, wine, yeast, non-*Saccharomyces*, *Saccharomyces cerevisiae*, malolactic bacteria, safety, quality

During the vinification process, we can generally separate four main phases associated with specific microbial dominances: (i) first stages of alcoholic fermentation (AF) (non-*Saccharomyces*), (ii) most part of AF, up to the end (*Saccharomyces*), (iii) malolactic fermentation (MLF) (lactic acid bacteria or LAB), and (iv) undesired changes associated to microbial metabolism (spoilage yeasts and bacteria, microbial producers of toxic compounds). All these microorganisms can be found ecologically associated to grapevines and to the vineyard and, consequently, to the winery environment. Furthermore, it should be stressed that in some cases strains involved in the phases of pro-technological interest (AF and MLF), are even responsible of undesired production (e.g., off-flavors, compounds toxic for human health). These evidences, together with the needs for standardization, time-saving procedures and quality/safety improvements, led to the introduction of the starter cultures technologies in the wine industry. Selected strains from natural “micro-biodiversity” and/or from breeding program were selected in order to design starter cultures, in other words “a microbial preparation of large numbers of cells of at least one microorganism to be added to a raw material to produce a fermented food by accelerating and steering its fermentation process.”

The research topic “Microbiota of grapes: positive and negative role on wine quality” belongs to the Food Microbiology section and covers 19 contributes: 1 review, 2 mini-reviews, and 16 original research papers. As Topic Editors, we briefly report an overview of these contributes starting with microbial consortia associated to grapes and wines. Indeed, nine of the articles focused on the description of the microbial consortia associated with specific grapes and with the corresponding (uninoculated) musts and wines. The following two studies analyzed both eukaryotic and prokaryotic microorganisms as target. Salvetti et al. described the microbial communities associated with the Italian *Vitis vinifera* L. cv. *Corvina* grape berries, used for the production of unique wines, such as Amarone, at the end of the process of “traditional withering” or “accelerated withering.” Pinto et al. characterized the microbiota associated with the must from six different Portuguese wine appellations. The first phases and last stages of AF were used as target. Piao et al. investigated the bacterial community and their temporal succession during the fermentation of organically grown Riesling grapes. Moreover, six work focused only on the “eukaryotic side.” Wang et al. described fungal diversity in Spanish “Carignan” and “Grenache” grape must and during wine fermentation. Sipiczki analyzed the yeast communities and their interactions in overwintering grapes (mummified on vines) in the Tokaj wine region (Hungary-Slovakia). Vigentini et al. delved into yeast biodiversity in five Georgian areas and from 22 different native cultivars using both grapes and wines. Padilla et al. examined yeast biodiversity from uninoculated fermentations from the Priorat region, the second “Denominación de Origen Calificada” wine region in Spain, while Aponte and Blaiotta surveyed yeasts diversity and enological significance in spontaneous fermentation from Taurasi DOCG (Appellation of Controlled and Guaranteed Origin) production area. Jara et al. observed the biodiversity of non-*Saccharomyces* yeast associated with vineyards of the in Chilean valleys. All these scientific reports provide us snapshot of microbial biodiversity throughout different methodological lenses: whole metagenome sequencing (Salvetti et al.; Piao et al.; Pinto et al.; Wang et al.), quantitative PCR and DGGE (Wang et al.), cultural-dependent methods followed by molecular characterization (Sipiczki; Vigentini et al.; Padilla et al.; Aponte and Blaiotta; Jara et al.).

The grape-associated microbial communities continuously change during the winemaking process, with different dominances that correspond to the main biotechnological steps that take place in wine. With concern of this succession, the special issues reported eight studies dealing with yeast characterization/applications and one concerning simultaneous AF and MLF. Two original research papers and a review article focused on the role of *Saccharomyces* strains. Patrignani et al. proposed a non-conventional characterization including release of volatile and, particularly, of sulfur compounds, of 10 *S. cerevisiae* strains inoculated in “Trebbiano” must. Capece et al. studied the diversity of indigenous *S. cerevisiae* strains associated with geographical origin from two different Italian wine-producing regions (Tuscany and Basilicata), in order to contribute to assess the possible role of these yeasts in the regional identity of wine. Legras et al. reviewed the most recent “omics” data on the analysis of flor strains of *S. cerevisiae*, an interesting phenotype for the aging of Sherry and Sherry-like wines. On the other hand, in accordance with the recent trends regarding the use of non-*Saccharomyces* in enology, five contributes reported literature review and original data on the use of specific species/strains to improve wine quality. Ciani et al. provided a review on the explored interactions among yeast species and strains of enological interest, with a particular focus on the effect of mixed cultures on the final wine quality, which can concretely influence the stability of the final wine and its analytical and aromatic profile. Grangeteau et al. demonstrated, for the first time, the persistence of non-*Saccharomyces* yeasts (*Hanseniaspora* and *Starmerella*) from year to year in the cellar. The work by Tristezza et al. reported new insights into the oenological potential of autochthonous Apulian strains of *Hanseniaspora uvarum* and *S. cerevisiae* used in simultaneous and sequential co-fermentation for industrial wine production. Tofalo et al. tested indigenous strains of *S. cerevisiae, Starmerella bacillaris*, and *H. uvarum* and a co-culture of *S. cerevisiae* and *S. bacillaris* to evaluate their role in the sensory characteristic of Montepulciano d'Abruzzo wine. Canonico et al. evaluated the use of specific immobilized non-*Saccharomyces* yeasts, in sequential fermentation, in order to reduce ethanol tenor in wine. With concern of MLF, the original research paper by Bleve et al. reported the efficacy of simultaneous alcoholic and malolactic fermentations by *S. cerevisiae* and *Oenococcus oeni* cells co-immobilized in alginate beads.

Finally, Russo et al. delved into safety aspect with a review on biogenic amines and mycotoxins, among the principal toxic compounds of microbial origin in wine, offering a brief description of the main determinant involved in this phenomena, but also overviewing the prevention/correction strategies, including those biotechnological-based.

In general, several paper contribute to improve the knowledge on the shape of autochthonous microbiota and on the significance of autochthonous yeasts for different geographical enological productions, in other terms on the so-called “microbial terroir,” a field that has been received considerable attention in last years.

Finally, this collection gives a flavor of the enological significance of the micro-biodiversity from grape to wine, highlighting in microbial resources the presence of a dichotomy: in each consortia there are species/strains that, in reason of their metabolisms, are able to improve wine “qualities” (resource of interest in starter cultures design), and species/strains that, with their metabolism, are responsible of depreciation of wine.

## Author contributions

GS and ST drafted and revised the final version of the Editorial.

## Funding

This work was supported in the framework of the projects named “Biotecnologie degli alimenti per l'innovazione e la competitività delle principali filiere regionali: estensione della conservabilità e aspetti funzionali (BiotecA)” and “Innovazioni di processo e di prodotto nel comparto dei vini spumanti da vitigni autoctoni pugliesi”—IPROVISP (Bando “Aiuti a Sostegno Cluster Tecnologici Regionali”; Project code VJBKVF4).

### Conflict of interest statement

The authors declare that the research was conducted in the absence of any commercial or financial relationships that could be construed as a potential conflict of interest.

